# 

**Published:** 1882-06

**Authors:** 


### AMERICAN MEDICAL ASSOCIATION.

The thirty-third annual meeting of the American Medical Association will be held at St. Paul, Minnesota, commencing Tuesday, June 6, and lasting four days. A section on dentistry was formed in this association at its last meeting, and medically educated dentists were recognized as specialists in medical science. All medical men of the regular school, practicing the specialty of dental surgery, are most cordially invited to procure credentials from their local medical societies and join us at St. Paul. All railroads furnish reduced rates to those members wishing to attend. "A member desiring to read a paper before any section should forward the paper or its title and length (not to exceed twenty minutes in reading) to the Chairman of the Committee of Arrangements at least one month before the meeting." (By-laws.)

TRUMAN W. BROPHY,
Secretary Section on Dentistry, American Medical Association.

CARLYLE once rode sixty miles to Edinburgh, to consult a doctor; "having," as he said, "reduced my perplexities to a single question: Is this disease curable by medicine? or is it chronic, incurable, except by regimen, if even so? This question I earnestly put, and got the response, 'It is all tobacco, sir; give up tobacco.' Gave it instantly and strictly up. Found, after long months, that I might as well have ridden sixty miles in the opposite direction, and poured my sorrows into the long, hairy ear of the first jackass I came upon, as into this medical man's, whose name I will not mention."

A WOMAN'S MEDICAL COLLEGE has just been started in Baltimore. All the professors are men.

### THE INDIANA STATE DENTAL ASSOCIATION.

The annual meeting of the Indiana State Dental Association will be held in the city of Indianapolis commencing the last Tuesday of June. All members of the dental and medical professions are cordially invited to attend.

The following programme has been sent to all members of the society with the request that they select the subject under which they wish their names to appear, and report to the Executive Committee.

PROGRAMME OF EXERCISES.

1st. Mechanical Dentistry.

*a.* Construction of Sets of Teeth with the New Mode Heater. *b.* Models Illustrating Articulation of Artificial Teeth, with a Paper.

2d. Operative Dentistry.

*a.* Preparation of Cavities. *b.* Different Kinds of Gold and their Uses.

3d. Pivot Teeth and Porcelain Sections.

*a.* Setting of Bonwill Crown. *b.* Restoring Contour with Porcelain Sections.

4th. Chemistry.

*a.* Acids and their Effects as Manifested Upon the Dental Organs. *b.* Paper by Prof. J. N. Hurty, of Indianapolis.

5th. Pathology and Therapeutics.

6th. Plastic Fillings, Permanent and Temporary.

7th. Dental Education and Literature.

8th. The Indiana State Dental Association.

EVENING SESSIONS.

1st Evening.

Development of Teeth. Lantern Illustrations.

2d Evening.

Regulation of Teeth with Models and Plates.

Clinics in the Infirmary of the Indiana Dental College.

J. B. MORRISON, Chairman Executive Committee.

### MISSOURI STATE DENTAL ASSOCIATION.

The eighteenth annual meeting of this body will be held at Sweet Springs, on Tuesday, June 6, at 10 o'clock, A. M.

The following programme for the occasion has been prepared:

1. Progress of Dentistry.
2. Children's Teeth.
3. Mercury and its Effects.
4. Tumors in the Oral Cavity.
5. Dental Legislation.
6. Dental Ethics.
7. Irregularities of the Teeth, with Appliances for Correcting.
8. High Civilization not a Cause of Tooth Decay.
9. The Treatment of the Cervical Wall.

Upon each of these subjects a paper, or papers, will be prepared by members of the Association. It is confidently expected that there will be a large attendance, and an interesting meeting. A number of dentists from other States will be present and take part in the proceedings. It is much to be desired that the members of the profession in the State should be present and aid in giving efficiency to the work.

### KENTUCKY STATE DENTAL ASSOCIATION.

The twelfth annual meeting of this association will be held in the rooms of the Polytechnic Society (which have been kindly tendered for our use) at Louisville, Ky., Tuesday, June 6, 1882, at 2:30 o'clock, P. M.

### SUBJECTS FOR DISCUSSION.

1. "Is there Continuity of Nutrition between Dentine and Cementum," A. W. Smith, J. S Cassidy.

2. "Sympathetic Neuralgia, so called," C. G. Edwards, A. O. Rawls.

3. "Method of Mounting Crowns on Roots of Teeth," F. Peabody, R. C. Morgan.

4. "Teeth Inserted Without Plates," J. F. Canine, J. B. Kidd.

5. "Chemical Conditions of Saliva during Gestation and Lactation," J. S. Cassidy, T. Y. Cooper.

6. "The Principles of the Action of Therapeutic Agents on Nerve Tissue," J. T. McMillan, J. Hooper.

7. Miscellaneous, J. M. Clyde, P. Burgin.

The Executive Committee respectfully urge a full attendance of the members.

A cordial invitation is extended to all in our profession to attend these meetings.

### ARRANGEMENTS FOR THE MEETING OF THE AMERICAN DENTAL ASSOCIATION.

The sessions of the meeting will be held in the hall of the Highland House, on one of the beautiful hill-tops for which Cincinnati is noted. This hall is about five hundred feet above the plain of the city, far from the heat, dust, and noise. It is ample, and has every convenience for the meeting that can be desired.

All the railroads entering the city have agreed to give reduced rates to those attending the meeting. The fare proposed is two cents per mile for the round trip The precise method of procuring tickets for those coming to the meeting will be given in the July REGISTER, and also by a circular that will be issued in due time. Very liberal reduction in hotel rates, and first-class accommodations have been prepared for those attending the meeting. The Gibson House, on Walnut Street, between Fourth and Fifth Streets, will probably be headquarters for the members.

The next number of the REGISTER will contain a more definite statement upon these matters. In the meantime, more definite information, if desired, can be obtained by addressing

J. TAFT, *Chairman of Local Committee.*

### CONTENTS.

### COMMUNICATIONS.

### THE DENTAL REGISTER,

A MONTHLY MAGAZINE DEVOTED TO THE INTERESTS OF THE

### DENTAL PROFESSION.

*Terms Reduced to $2.00 Per Year. Single Copies, 25 Cents.*

### EDITED BY PROF. J. TAFT, D.D.S.

—AND—

### *Published by SPENCER & CROCKER, Ohio Dental Depot.*

The volume commences in January and closes in December.

The REGISTER being issued monthly is always fresh, therefore Indispensable to the practitioner who desires to keep posted up with the new inventions and improvements which are daily developed. Neither expense nor effort will be spared to give value and variety to its contents. Articles will be illustrated with well executed engravings whenever they will add to the interest or assist to explanation.

Its extensive circulation and frequency of issue make it one of the BEST DENTAL ADVERTISING MEDIUMS in the United States.

All subscriptions must begin with January or July.

Local or special notices, five lines or less, will be inserted for $3 each insertion; over five lines, 50 cts. per line.

All advertising bills payable in advance, or at furthest, after the issue of the first number containing the advertisement. All transient advertisements to be paid for in advance.

☞CONTRIBUTIONS SOLICITED.

Exclusively Editorial Communications should be addressed to the Editor.

The latest number of the REGISTER may be ordered with or without other goods at any time, and all letters should be addressed to

**SPENCER & CROCKER, Ohio Dental Depot, Cincinnati, Ohio.**

